# Changes in Locomotor Activity and Oxidative Stress-Related Factors after the Administration of an Amino Acid Mixture by Generation and Age

**DOI:** 10.3390/ijms22189822

**Published:** 2021-09-10

**Authors:** Yejin Ahn, Ki-Bae Hong, Suhyeon Kim, Hyung Joo Suh, Kyungae Jo

**Affiliations:** 1 Department of Integrated Biomedical and Life Science, Graduate School, Korea University, Seoul 02841, Korea; ahnyj708@gmail.com (Y.A.); sh_kim3@lotte.net (S.K.); 2 Department of Food Science and Nutrition, Jeju National University, Jeju 63243, Korea; kbhong@jejunu.ac.kr; 3 Lotte R&D Center, Seoul 07594, Korea; 4 Transdisciplinary Major in Learning Health Systems, Department of Healthcare Sciences, Graduate School, Korea University, Seoul 02841, Korea

**Keywords:** γ-aminobutyric acid, 5-hydroxytryptophan, sleep, reactive oxygen species, generation, age

## Abstract

Amino acids, as nutrients, are expected to improve sleep disorders. This study aimed to evaluate the generation- and age-dependent sleep-improving effects of γ-aminobutyric acid (GABA) and 5-hydroxytryptophan (5-HTP) coadministration. The differentially expressed genes and generation-related behavior after the administration of a GABA/5-HTP mixture were measured in a *Drosophila* model, while age-related changes in gene expression and oxidative stress-related parameters were measured in a mouse model. The GABA/5-HTP-treated group showed significant behavioral changes compared to the other groups. Sequencing revealed that the GABA/5-HTP mixture influenced changes in nervous system-related genes, including those involved in the regulation of the expression of behavioral and synaptic genes. Additionally, total sleep time increased with age, and nighttime sleep time in the first- and third-generation flies was significantly different from that of the control groups. The GABA/5-HTP mixture induced significant changes in the expression of sleep-related receptors in both models. Furthermore, the GABA/5-HTP mixture reduced levels of ROS and ROS reaction products in an age-dependent manner. Therefore, the increase in behavioral changes caused by GABA/5-HTP mixture administration was effective in eliminating ROS activity across generations and ages.

## 1. Introduction

Circadian rhythm is an essential component and a universal phenomenon of the health of every animal, from mammals to insects, studied so far. A stable cycle of restorative rest is necessary for humans to perform normal activities because circadian rhythm dysfunction has been shown to lead to fatigue, aggression, and anxiety [1]. Without patterns in the behavior and physiology, humans experience a loss of energy and vitality, and a variety of health problems arise. Although circadian rhythm dysfunction can be treated pharmacologically, many side effects, such as tolerance and dependence, have been reported with the long-term use of pharmacological treatment [2]. In addition, medications commonly prescribed for a variety of diseases are known to affect circadian rhythm disturbances such as decreased sleep quality and altered sleep architecture [3].

In previous studies, the inhibitory effects of a γ-aminobutyric acid (GABA)/5-hydroxytryptophan (5-HTP) mixture have been identified in both invertebrate and vertebrate models [4,5,6]. Indeed, the mixture of GABA and 5-HTP shows synergistic effects on reduction of total activity in a *Drosophila* model. Similarly, the GABA/5-HTP mixture increases nonrapid eye movement (NREM) sleep and sleep duration in ICR mice, Sprague–Dawley rats, and a caffeine-induced sleepless model. However, as these mixtures have only been evaluated for activity in single animals or in single generations of animals, differences in the long-term use or age-specific activities of the mixture are worth examining.

Deep NREM sleep shows marked changes during life, which are closely associated with aging; NREM sleep peaks at puberty and declines thereafter [7,8,9,10]. These findings suggest that the aging process may be associated with the features of sleep, as the percentage of NREM sleep decreases with age [9,10]. Reactive oxygen species (ROS) that accumulate during awake periods are removed during sleep [11]. Uncontrolled ROS production under sleep deprivation is also known to contribute to the development of various diseases, such as circadian rhythm disturbances and sleep apnea [12,13]. While awake, oxygen in the brain is primarily used for metabolism, such as electron transfer in the nervous system, causing an increase in ROS levels. During sleep, antioxidant activity is enhanced to protect the brain from ROS. ROS are reportedly associated with sleep deprivation processes [11,14]. Sleep has been shown to reduce oxidative stress and is involved in repair and detoxification processes [15].

In this study, GABA, 5-HTP, and a GABA/5-HTP mixture were used to reduce behavior in a *Drosophila* model. Using a locomotor activity assay, we identified that these two amino acids and their mixture regulate the total activity during subjective nighttime and daytime. The inhibitory effects of the GABA/5-HTP mixture were confirmed by high-throughput RNA sequencing, and the genes that were related to these effects were validated. In addition, the effects on locomotor activity- and stress-related factors were also investigated following age-related or intergenerational administration of the GABA/5-HTP mixture.

## 2. Results

### 2.1. Locomotor Activity

**Figure 1:**
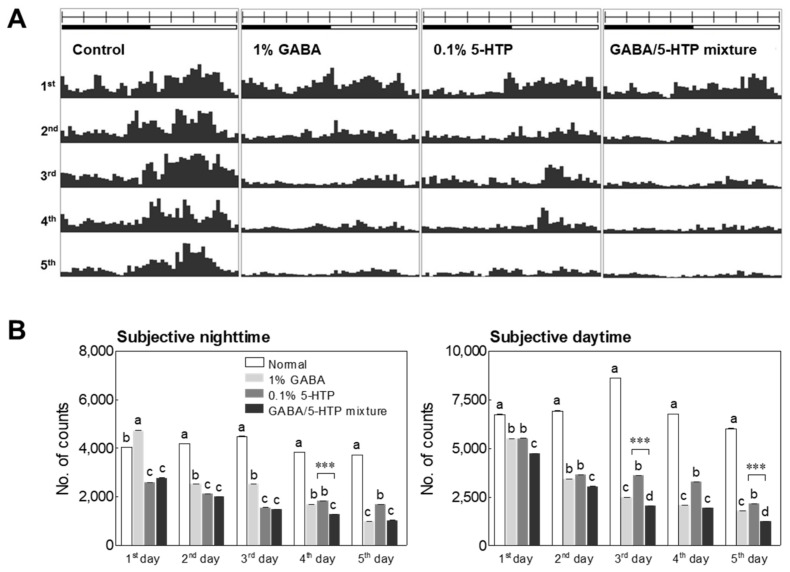
Effects of the two amino acids and the GABA/5-HTP mixture on the locomotor activity of Drosophila flies. (**A**) Typical actograms of the control group (*n* = 100) and flies exposed to GABA (*n* = 100), 5-HTP (*n* = 100), and the GABA/5-HTP mixture (*n* = 100). The graphic representation of locomotor activities was generated by the Actogram J program using average activity in a 30 min interval calculated over 5 days. (**B**) Locomotor activities during subjective nighttime and daytime. Data are presented as means ± standard error of the mean for each group. Bars marked with different letters indicate significant (*p* < 0.05) differences between the time points from each other by Tukey’s test. Symbols indicate significant differences at *** *p* < 0.001 by two-way ANOVA analysis.

The locomotor activity of *Drosophila* was evaluated to assess the inhibitory effects of GABA, 5-HTP, and the GABA/5-HTP mixture. Figure 1A shows the actogram of the *Drosophila* motor activity of all groups for five days using the locomotor activity monitoring (LAM) system. Figure 1B shows the behavioral changes during the subjective nighttime and daytime for five days. The locomotor activity during the dark phase of days 1, 2, and 3 was significantly lower in the 0.1% 5-HTP-treated and GABA/5-HTP-treated groups than in the control group (*p* < 0.01 and *p* < 0.001), and the behavior from day 2 was significantly lower in the 1% GABA-treated group than the control group (*p* < 0.01). Treatment groups (1% GABA, 0.1% 5-HTP, and the GABA/5-HTP mixture) showed significantly different locomotor activity compared to that of the control group (*p* < 0.01) on day 4 (*p* < 0.05). The dark phase activity of day 5 was also significantly lower in the GABA-, 5-HTP-, and GABA/5-HTP-treated groups (*p* < 0.001) than in the control group. The locomotor activity during the light phase of the total experimental period significantly decreased with GABA/5-HTP mixture administration (*p* < 0.01 and *p* < 0.001). In addition, two-way ANOVA with repeated measures revealed that the GABA/5-HTP mixture had an interactive effect on the dark phase activity of day 4 and the light phase activity of days 3 and 5 (*p* < 0.001). Results showed that the immediate and stable effects of both amino acids in group activities are compounded in the GABA/5-HTP mixture.

### 2.2. Transcriptome Sequencing and Differential Expression Analysis

As a result of RNA sequencing, single-end raw reads were generated, and approximately 70% of the reads were mapped to *Drosophila* reference mRNA sequences. The number of differentially expressed genes (DEGs) that satisfied |fold change| ≥ 4 (FC4) or |fold change| ≥ 2 (FC2) is shown in Table 1. To screen the DEGs following GABA/5-HTP treatment, FCs were calculated by comparing the expression of identified transcriptomes, and 101 (FC4) and 646 (FC2) genes that were biologically significant were detected. Of these DEGs, 56 and 342 were upregulated, respectively, and 45 and 304 were downregulated, respectively, by GABA/5-HTP administration.

The gene network construction and function prediction of FC4 and FC2 genes are summarized in Table 2, Table 3 and Table 4. In general, the fragments per kilobase of exon per million fragments mapped (FPKM) values of the transcriptome were related to various cellular components, biological processes, and molecular functions. The FPKM values of FC4 and FC2 genes related to GABA synthesis, release, reuptake, and degradation were significantly different through the administration of GABA/5-HTP mixture. In addition, GABA/5-HTP administration was related to the regulatory system processes, synaptic vesicles, neurotransmitter release cycles, and neuronal system.

### 2.3. Gene Function Prediction

**Figure 2:**
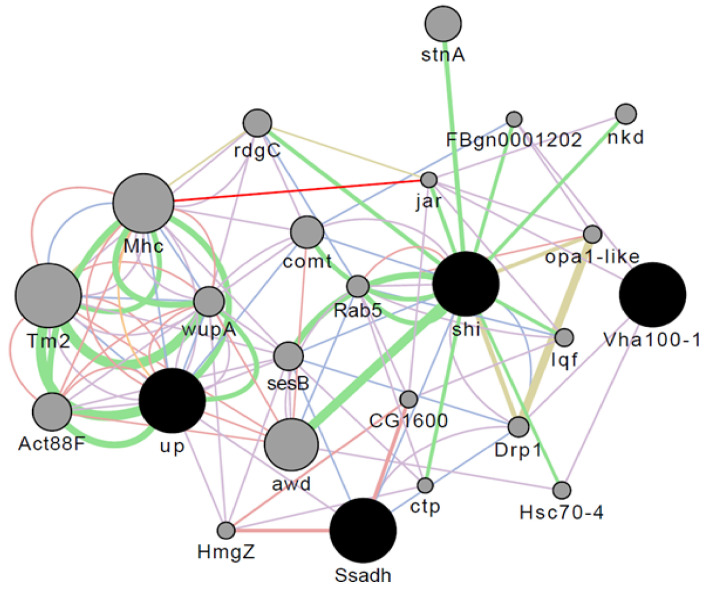
Composite network of multiple regulation of the GABA/5-HTP-treated group with differentially expressed genes that satisfied |fold change| ≥ 2. The black circles are downregulated genes, and 11 most related genes and 20 most related attributes are shown. The source networks are grouped by type network weight, as well as the sum of the weight of the networks in the group.

The gene succinate-semialdehyde dehydrogenase (*Ssadh*) was significantly more expressed in the GABA/5-HTP-treated group than in the control group (Figure 2). *Ssadh* is involved in the GABA catabolic process and is closely related to the neuronal system, including the neurotransmitter release cycle and transmission. The GABA catabolic process is an important biological process, and *Ssadh* is colocalized with *sesB*. *sesB* is coexpressed with other genes and is involved in muscle cell cellular homeostasis and genetic interactions with various other genes. *Mhc* is related to flight behavior and directly interacts with the *jar* gene. These results indicate that the GABA/5-HTP treatment significantly affected genes closely related to behavior.

### 2.4. Locomotor Activity in Drosophila Flies by Generation

**Figure 3:**
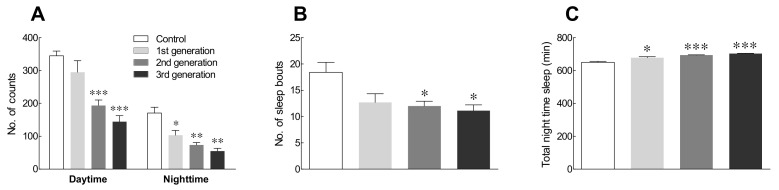
Effects of the GABA/5-HTP mixture (1.0% and 0.1%, respectively) on the locomotor activity of Drosophila flies by generation. Behavioral analysis was performed under constant darkness (DD) for five days. (**A**) Subjective nighttime activity, (**B**) number of sleep episodes, and (**C**) subjective nighttime sleep duration, as measured using the Drosophila activity monitoring system. Activity was measured as counts per min and calculated over five days. Data are presented as means ± standard error of the mean for each group. Mean values with different letters over the bars indicate significant difference (* *p* < 0.05, ** *p* < 0.01, and *** *p* < 0.001) according to Student’s *t*-test.

GABA/5-HTP administration tended to decrease total locomotor activity as the generations progressed, but there was no significant difference in the locomotor activity between generations (Figure 3A). Sleep bouts were not significantly different between the first generation of GABA/5-HTP mixture-treated flies and the control flies. However, there was a significant difference after the second generation compared to the control flies (Figure 3B; *p* < 0.05). Total nighttime sleep time increased with generation, and it was significantly different between the control and GABA/5-HTP-treated groups (Figure 3C; *p* < 0.05 and *p* < 0.001). Overall, sleep-related activity tended to increase as the generation increased, without the development of tolerance.

### 2.5. Receptor Expression in Drosophila Flies by Generation

**Figure 4:**
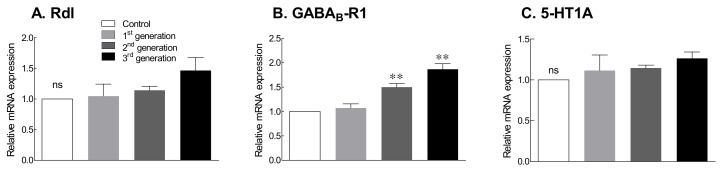
Effects of the GABA/5-HTP mixture (1.0% and 0.1%, respectively) on Rdl (**A**), GABA_B_-R1 (**B**), and 5-HT1A (**C**) mRNA expression in Drosophila flies by generation. Data are presented as means ± standard error of the mean for each group. Mean values with different letters over the bars indicate significant difference (** *p* < 0.01) according to Student’s *t*-test. ns, not significant.

Drug tolerance that develops owing to long-term administration of benzodiazepines, used as sleeping agents, is caused by a decrease in the levels of GABA receptors. Therefore, the receptor expression was measured to evaluate drug tolerance to the GABA/5-HTP mixture across generations (Figure 4). The expression of the GABA_A_ receptor (resistance to dieldrin: Rdl) increased with increasing generations, but the difference was not statistically significant (Figure 4A). GABA_B_-R1 receptor expression tended to increase with increasing generations and was significantly different in the second and third generations compared with that in the control group (Figure 4B; *p* < 0.01). 5-HT1A receptor expression also tended to increase with increasing generations, but the difference from the control group was not significant (Figure 4C). The expression of the receptor, one of the indicators of drug tolerance, increased with increasing generation, and it is suggested that no tolerance to the GABA/5-HTP mixture was developed.

### 2.6. ROS Production in Drosophila Flies by Generation

**Figure 5:**
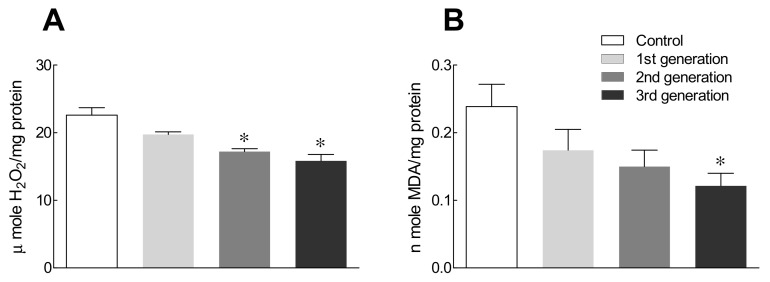
Effects of the GABA/5-HTP mixture (1.0% and 0.1%, respectively) on the amount of hydrogen peroxide (**A**) and lipid peroxidation (**B**) in Drosophila fly heads across generations. Data are presented as means ± standard error of the mean for each group (*n* = 50 per group). The different letters indicate significant (* *p* < 0.05) differences from the control group by Student’s *t*-test. H_2_O_2_, hydrogen peroxide; MDA, malondialdehyde.

Endogenous ROS produced from aerobic metabolism may be the most important cause of nerve damage. In addition, ROS have been reported to be strongly associated with insomnia. Thus, we examined the effects of the GABA/5-HTP mixture on ROS production over generations as ROS can affect brain cell damage (Figure 5). The amount of hydrogen peroxide (H_2_O_2_) was significantly lower in the second and third generations of the GABA/5-HTP-treated group compared to the control group (Figure 5A; *p* < 0.05). The amount of malondialdehyde (MDA) tended to decrease with each generation in the GABA/5-HTP-treated group and was significantly lower in the third generation compared to the control group (Figure 5B). Collectively, GABA/5-HTP administration helped reduce ROS production over generations.

### 2.7. Age-Related Receptor Expression in Mice

**Figure 6:**
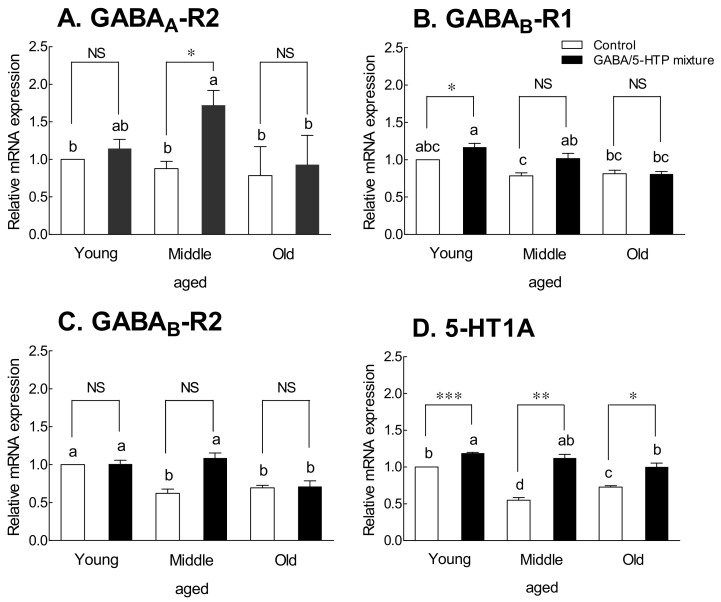
Effects of the GABA/5-HTP mixture (60 and 6 mg/kg, respectively, oral administration for 14 days) on age-related changes in mRNA expression of receptors: GABA_A_ receptor (**A**), GABA_B_ receptor 1 (**B**), GABA_B_ receptor 2 (**C**), and 5-HT1A receptor (**D**). Young (6 weeks), middle-aged (6 months), and old (1 year) mice were used. Data are presented as means ± standard error of the mean for each group (*n* = 5 per group; control: 0.9% physiological saline group). The different letters indicate significant (*p* < 0.05) differences from the control group by Tukey’s test. Symbols indicate significant differences at * *p* < 0.05, ** *p* < 0.01, and *** *p* < 0.001 as compared with the control group by Student’s *t*-test. NS, not significant.

The expression of GABA receptors in each age group after oral administration of the mixture for 14 days was measured (Figure 6). GABA_A_-R2 and GABA_B_-R1 receptor expression in the control group did not differ with age (Figure 6A,B). However, there was a significant difference in the expression of GABA_A_-R2 receptors at middle age and GABA_B_-R1 receptors at young age between the GABA/5-HTP-treated group and the control group (*p* < 0.05). The expression of GABA_B_-R2 and 5-HT1A receptors decreased with age (Figure 6C,D, *p* < 0.05). In the GABA/5-HTP-treated group, the expression of 5-HT1A receptors was significantly different across age-matched controls, and the difference was significant at young age (Figure 6D; young-aged, *p* < 0.001; middle-aged, *p* < 0.01; old-aged, *p* < 0.05).

### 2.8. Age-Related ROS Production in Mice

**Figure 7:**
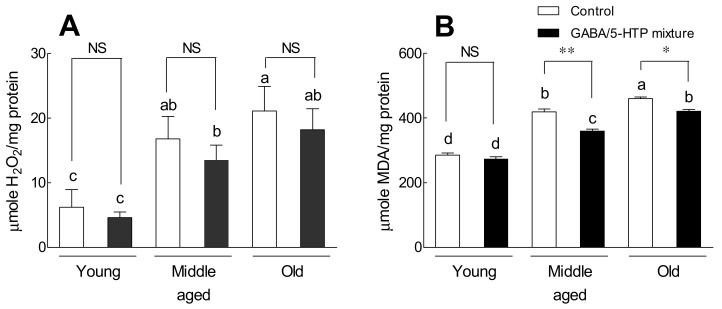
Effects of the GABA/5-HTP mixture (60 and 6 mg/kg, respectively, oral administration for 14 days) on age-related changes in the amount of hydrogen peroxide (**A**) and lipid peroxidation (**B**) in mouse brains. Young (6 weeks), middle-aged (6 months), and old (1 year) mice were used. Data are presented as means ± standard error of the mean for each group (*n* = 5 per group; control: 0.9% physiological saline group). The different letters indicate significant (*p* < 0.05) differences from the control group by Tukey’s test. Symbols indicate significant differences at * *p* < 0.05 and ** *p* < 0.01 as compared with the control group by Student’s *t*-test. NS, not significant.

Figure 7 shows the effect of the GABA/5-HTP mixture on the amount of H_2_O_2_ and MDA, the ROS product, in mice of different ages. With aging, there was a significant increase in the amount of H_2_O_2_ and MDA (Figure 7). The GABA/5-HTP-treated groups had a considerably lower amount of H_2_O_2_ than the corresponding control groups (Figure 7A). On the other hand, the amount of MDA in the GABA/5-HTP-treated group was not significantly different from that in the control group at young age but was significantly lower in middle-aged and old-aged mice (Figure 7B; *p* < 0.01 and *p* < 0.001, respectively).

## 3. Discussion

Most medicines prescribed for insomnia carry the risk of overdose, tolerance, habituation, and addiction. Natural products are widely used as sedatives to improve brain inhibitory mechanisms and prevent side effects, including cognitive impairment, tolerance, and addiction disorders [16]. GABA is a major inhibitory neurotransmitter in humans and animals and is composed of a non-protein amino acid [17]. The main benefits of GABA administration are calm, relaxation, and stress reduction. GABA supplements are also one of the most popular natural solutions for better sedative effect. The effect of GABA is mediated through the interaction with GABA_A_ receptors, which causes the opening of chloride ion-selective pores and a subsequent increase in chloride flux [18,19]. Five-HTP is present in large amounts in the seeds of *Griffonia simplicifolia*, which is of West African origin [20]. Also, 5-HTP is a precursor to serotonin, which is responsible for regulating mood and circadian rhythms and is also indirectly involved in the production of melatonin, an important sleep-related hormone [21]. However, no previous study has investigated the tolerance and behavioral patterns of a GABA/5-HTP mixture across generations or ages.

In *Drosophila* and mammals, GABA and serotonin play a similar role; that is, they have sustained inhibitory effects. GABA promotes normal sequencing of circadian behavioral rhythms and reduces arousal through ionic and metabolic receptors [22,23]. Serotonin also promotes neuronal inhibitory signaling through the d5-HT1A receptor and affects circadian rhythm [24].

In the present study, we demonstrated the time-dependent effects of the two amino acids and the GABA/5-HTP mixture on behavioral changes during subjective nighttime and daytime using the LAM system (Figure 1). These amino acids are important neurotransmitters in the mammalian circadian pacemaker neurons. The GABA/5-HTP mixture has a dose-dependent inhibitory effect in invertebrate and vertebrate models [4,5]. Mehling et al. [25] performed a robust normalization of quantitative polymerase chain reaction (qPCR) data from human blood to identify potential biomarkers of γ-hydroxybutyric acid, an agonist of the GABA_B_ receptor and a medication for narcolepsy. In the serotonergic system, Egr3, tryptophan hydroxylase (Trh in flies and Tph in mammals), and norepinephrine/serotonin uptake transporter (Net and Sert) genes play an essential role in regulating cortical arousal and sleep [26,27,28].

To identify gene expression changes induced by GABA/5-HTP administration, RNA sequencing was performed. Additionally, to elucidate the action mechanism of the GABA/5-HTP mixture, we conducted DEG analysis and used the DAVID functional annotation tool for functional enrichment analyses (Table 2, Table 3 and Table 4). The *sesB* gene has transmembrane transporter activity and is involved in the biological process in response to stimulus, cellular response to chemical stimuli, locomotor behavior, action potential, locomotion, and response to external stimuli [29,30,31]. Moreover, *sesB* interacts genetically with *Rab5*, which is related to the regulation of biological processes, multiorganism processes, biological quality, and cellular responses to stimuli [32,33,34]. The Shibire (*shi*) gene is colocalized with *sesB* and interacts genetically with *jar*. Moreover, *shi* is involved in the biological processes of synapses, learning, and memory [35,36].

Behavioral patterns after administration of GABA/5-HTP mixture for each generation showed a decrease in behavior with increasing generation (Figure 3). In particular, the amount of GABA_A_-R (Rdl) receptors tended to increase nonsignificantly when compared with the expression levels of the receptors between generations (Figure 4). Similarly, the RNA levels of GABA_B_-R1 and 5-HT1A receptors also increased in each generation (Figure 4). Our previous study [4] also reported an increase in the expression of GABA_B_-R1 and 5-HT1A receptors. Ninan [37] demonstrated that the tolerance to benzodiazepine after long-term administration is due to a decrease in the amount of GABA_A_ receptors. Therefore, we deduced that no tolerance developed because of the intergenerational administration of the GABA/5-HTP mixture based on circadian rhythm-related behavior and the expression of the GABA_A_ receptors. The inhibitory effects of the GABA/5-HTP mixture in each generation were considered to be owing to GABAergic and serotonergic neurons, although there was no significant difference between generations.

Disturbances or imbalances in the relationship between circadian and homeostatic systems can lead to sleep disturbances. Even in the absence of clinical sleep disturbances, aging in healthy individuals is associated with decreased sleep quality, sleep duration, and sleep continuity [38]. Elderly people are at risk of sleep disturbances because of age-related psychological and physiological changes. Changes in sleep patterns and circadian rhythms are a part of normal aging processes, such as physical changes that occur with age. Aging alters the structure of sleep and affects the increase in spontaneous arousal that often occurs in older people [39]. Also, sleep structure, including sleep latency and total sleep duration, i.e., quantity and quality of sleep, are all affected by aging [40].

GABA_A_ receptors are important for drug action and regulation of benzodiazepines, barbiturates, and steroids in the mammalian central nervous system [41]. Changes in receptors are particularly important in the aging of the central nervous system [42] and may be responsible for some of the sleep pattern changes that are affected by aging. However, reports on age-related changes in GABA_A_ receptors have been inconsistent [43]. Some researchers [44] demonstrated an increase in specific mRNA or protein levels of certain subunits, while others have reported no changes [19]. As shown in Figure 6, mRNA expression of GABA_A_-R2 and GABA_B_-R1 receptors did not change regardless of age, whereas that of GABA_B_-R2 and 5-HT1A receptors tended to decrease with age. In particular, 5-HT1A expression was significantly different from that in the control group in each age group. This seems to be because of the effect of 5-HTP in the GABA/5-HTP mixture. Several studies have indicated that 5-HTP improves sleep patterns by enhancing melatonin synthesis [45,46].

Furthermore, our results showed that behavioral changes were associated with a greater amount of H_2_O_2_ and MDA in old-aged mice than in young and middle-aged mice. Additionally, old-aged brain regions were more severely affected by ROS (Figure 5 and Figure 7). Continuous generational administration of the GABA/5-HTP mixture resulted in a decrease in the amount of H_2_O_2_ in the *Drosophila* model (Figure 5) and an age-related decrease in the amount of MDA, a lipid peroxidation product, in the mouse model (Figure 7). Melatonin and the serotonin precursor 5-HTP can remove radicals and combat lipid peroxidation owing to their antioxidant action [47]. Keithahn and Lerchl [48] reported that 5-HTP is more effective as an in vitro hydroxyl radical scavenger than either melatonin or ascorbic acid. MDA-related carbonyl stress damages neurons via Ca^2+^ influx and overload. GABA inhibits the formation of reactive carbonyl intermediates during oxidative stress and reacts with MDA to form different conjugated complexes in vitro [18]. These results demonstrate the potent function of the GABA/5-HTP mixture in ameliorating behavioral change or ROS production caused by aging.

## 4. Materials and Methods

### 4.1. Fly Stocks

Wild-type Canton-S strain flies were obtained from the Bloomington *Drosophila* Stock Center (Bloomington, IN, USA). *Drosophila* flies were propagated on standard cornmeal agar medium (sucrose, cornmeal, yeast, agar, propionic acid, and p-hydroxybenzoic acid methyl ester solution) in a breeding environment that was set for a 12 h day and night cycle in a chamber maintained at 25 °C and 60% humidity.

Male ICR mice (young age: 6 weeks old, middle age: 6 months old, old age: 1 year old) were supplied by Central Lab Animal, Inc. (Seoul, Korea), and used for experiments after acclimatization for 1 week. Mice were bred in an environment where constant temperature (23 ± 1 °C), humidity (approximately 60%), and day and night cycles (12 h each) were maintained, and food and water were provided ad libitum. Animal experiments were conducted in compliance with the ethical regulations and approval of the Korea University Animal Care Committee (KUIACUC-2018-53, Seoul, Korea).

### 4.2. Behavioral Assays

Behavioral analysis was conducted according to the method reported in a previous study [4]. Behavioral analysis was performed using the LAM system (TriKinetics, Waltham, MA, USA) when *Drosophila* flies ingested a sucrose agar medium containing 1% GABA, 0.1% 5-HTP, or GABA/5-HTP (1% GABA and 0.1% 5-HTP). The group (minimum 20 flies) activity, which was measured by the LAM system, provided locomotor activity levels. All experiments were repeated five times.

Changes in locomotor activity by generation were measured using a *Drosophila* activity monitoring system (DAM, TriKinetics), and these measurements involved housing *Drosophila* flies individually in glass tubes. *Drosophila* flies were acclimated to the tube for 24 h, and all recordings were measured with an infrared detector every minute during the experiment in constant darkness at 25 ± 1 °C. Data were analyzed using the Actogram J software, and sleep parameters were calculated by summing the number of all activities recorded daily. Subjective nighttime activity was calculated as the sum of total activity, and subjective nighttime sleep was calculated as the sum of total sleep in the light and dark phases. Sleep was defined as a period of inactivity for at least 5 min (0 interruptions per minute). The number of sleep bouts was calculated by adding the sleep episodes.

### 4.3. High-Throughput RNA Sequencing and DEG Analysis

High-throughput sequencing was performed using HiSeq 2000 (Illumina Inc., San Diego, CA, USA), and total RNA was isolated from the heads of control and GABA/5-HTP-treated *Drosophila* flies using an RNA extraction kit (RNeasy Mini Kit, Qiagen, Venlo, The Netherlands) according to the manufacturer’s instructions. The libraries were quantified using qPCR according to the qPCR Quantification Protocol Guide (KAPA Library Quantification Kits for Illumina sequencing platforms) and qualified using an Agilent Technologies 2100 Bioanalyzer (Illumina). To determine the expression and identify alternatively spliced transcripts, the RNA-Seq reads were mapped against the genome of *Drosophila melanogaster* using TopHat [49], the transcript counts at the isoform level and gene level were calculated, and the relative transcript abundances were converted and measured as FPKM values using Cufflinks [50].

Filtered data were logarithmically transformed and normalized using the quantile normalization method. For each transcript, the FC of the treatment versus control was calculated. The significant results by adjusting to FC2 or FC4 were finally determined. The GeneMANIA application in Cytoscape was used to analyze and visualize multifunctional genes [51,52].

### 4.4. mRNA Expression of Neurotransmitter Receptors

*Drosophila* flies were exposed to a medium containing the GABA/5-HTP mixture (0.1 and 1.0%, respectively) for 14 days (*n* = 50 per group), and mice were administered the GABA/5-HTP mixture (6 and 60 mg/kg, respectively) once a day for 14 days (*n* = 5 per group). RNA was isolated from fly heads and mouse brains using TRIzol reagent (Invitrogen, Carlsbad, CA, USA). Genomic DNA was removed using RQ1 RNase-free DNase I (Promega, Madison, WI, USA). cDNA was synthesized using oligo-d(T) primers and SuperScript III Reverse Transcriptase (Invitrogen). Gene expression was measured for 45 cycles at 94 °C for 20 s, 55 °C for 40 s, and 72 °C for 40 s using the AB7300 Real-Time PCR system (Applied Biosystems, Foster City, Calif) and SYBR green mixture. Quantitative analysis of the gene expression was determined after normalization to the expression of the reference genes *RpL32* (fruit fly: NM_001144655.3) and *β-actin* (mice: NM_007393.5) using the ∆∆Ct method [53].

### 4.5. Measurement of MDA and H_2_O_2_

The amounts of MDA and H_2_O_2_ in *Drosophila* heads and mouse brains were analyzed after 14 days of treatment with the GABA/5-HTP mixture (1.0, 0.1%; 60, 6 mg/kg, respectively). The amount of MDA was analyzed according to the modified method of Buege and Aust [54]. Briefly, the brain tissue was homogenized with cold Tris-HCl buffer (50 mM, pH 7.5) and the homogenate was centrifuged at 4000× *g* for 10 min at 5 °C to obtain a supernatant, which is the tissue homogenate. A reaction buffer (200 μL of 8.1% sodium dodecyl sulfate, 1.5 mL of 0.8% TBA, 1.5 mL of 20% acetic acid, and 0.6 mL of distilled water) was added to the tissue homogenate and incubated for 1 h at 95 °C. After cooling, the reaction mixture was added with butanol (3 mL), mixed, and centrifuged at 800× *g* for 10 min to obtain 200 μL of the supernatant. The absorbance was measured at 532 nm using a microplate leader (Tecan M200, Männedorf, Switzerland).

The amount of H_2_O_2_ produced because of oxidative stress was determined using an Oxi Tec assay kit (BIOMAX, Rockville, MD, USA). The absorbance was measured at Ex/Em of 540/590 nm. The amount of each component was expressed as the amount of protein in the sample.

### 4.6. Statistical Analysis

The experimental results are expressed as the means ± standard error of the mean. The statistical analyses were conducted using the SPSS Statistics 18 (IBM Corporation, Armonk, NY, USA) statistical package. A significant difference test for each sample group was analyzed by one-way analysis of variance followed by Tukey’s multiple range test. Student’s *t*-test and two-way ANOVA analysis were used for comparisons between two groups and comparisons between multiple groups, respectively. *p*-values < 0.05 were considered statistically significant.

## 5. Conclusions

Administration of the GABA/5-HTP mixture reduced ROS production and regulated locomotor activity in each generation, without tolerance developing, in the *Drosophila* model. In addition, the mixture modulated locomotor activity and reduced the levels of ROS reaction products with age. These effects appear to be due to changes in the expression levels of GABA receptors. The differences in the generation-related effects on the *Drosophila* model are presumed to be due to differences in the expression of GABA_B_-R1 and 5-HT1A receptors, and the age-related effects on the mouse model appear to be due to differences in 5-HT1A receptor expression. Collectively, these results confirmed that administration of the GABA/5-HTP mixture is effective in modulating locomotor activity and reducing ROS levels across generations and ages. Taken together, we demonstrated that the administration of an amino acid mixture induces behavioral changes by regulating the expression of genes related to neurotransmitter release cycle and transmission, as well as specific receptor expression. We also reported that the administration of the GABA/5-HTP mixture was associated with a decrease in ROS-related factors generated in the brain across generations and ages. However, the effects of GABA/5-HTP mixture on the antioxidant mechanism-related parameters were not confirmed, and additional research is necessary to elucidate the role of decreased locomotor activity through combined administration on oxidative stress-related mechanisms.

## Figures and Tables

**Table 1:** The number of differentially expressed genes that satisfied |fold change| ≥ 2 or |fold change| ≥ 4 in the *Drosophila* model.

	Differentially Expressed Genes in *Drosophila* after GABA/5-HTP Treatment		
Categories	Number of Transcripts		
Total	29,904		
Filtered gene	27,191		
|fold|≥ 4	101	Up	56
		Down	45
|fold|≥ 2	646	Up	342
		Down	304

**Table 2:** Cellular component groups identified by the functional annotation clustering analysis with |fold change| ≥ 4 and 2 (DAVID functional analysis).

**|Fold Change| ≥ 4 (DAVID Functional Analysis)**			
**GO Term**	**Gene Count**	***p*-Value**	**GO Accession**
nucleolus	7	2.07 × 10^−0.5^	GO:0005730
nuclear lumen	7	8.30 × 10^−3^	GO:0031981
intracellular organelle lumen	8	1.27 × 10^−2^	GO:0070013
organelle lumen	8	1.27 × 10^−2^	GO:0043233
membrane-enclosed lumen	8	1.46 × 10^−2^	GO:0031974
**|Fold Change| ≥ 2 (DAVID Functional Analysis)**			
**GO Term**	**Gene Count**	***p*-Value**	**GO Accession**
cell cortex	7	0.01	GO:0005938
nucleolus	8	0.04	GO:0005730

GO—gene ontology.

**Table 3:** Biological process groups identified by the functional annotation clustering analysis with |fold change| ≥ 4 and 2 (DAVID functional analysis).

**|Fold Change| ≥ 4 (DAVID Functional Analysis)**			
**GO Term**	**Gene Count**	***p*-Value**	**GO Accession**
phagocytosis, engulfment	4	0.01	GO:0006911
phagocytosis	4	0.01	GO:0006909
endocytosis	4	0.03	GO:0006897
membrane invagination	4	0.03	GO:0010324
membrane organization	4	0.04	GO:0016044
**|Fold Change| ≥ 2 (DAVID Functional Analysis)**			
**GO Term**	**Gene Count**	***p*-Value**	**GO Accession**
cellular ion homeostasis	7	2.21 × 10^−4^	GO:0006873
ion homeostasis	7	4.51 × 10^−4^	GO:0050801
regulation of membrane potential	5	4.75 × 10^−4^	GO:0042391
cellular chemical homeostasis	7	5.03 × 10^−4^	GO:0055082
chemical homeostasis	7	0.00	GO:0048878
cellular homeostasis	8	0.01	GO:0019725
homeostatic process	8	0.05	GO:0042592
response to abiotic stimulus	11	0.01	GO:0009628
visual behavior	3	0.04	GO:0007632
positive regulation of cellular component organization	6	1.42 × 10^−4^	GO:0051130
regulation of cellular component biogenesis	6	0.02	GO:0044087
glutamine family amino acid catabolic process	3	0.01	GO:0009065
glutamate metabolic process	3	0.02	GO:0006536
cellular amino acid catabolic process	4	0.03	GO:0009063
amine catabolic process	4	0.03	GO:0009310
glycerol metabolic process	3	0.05	GO:0006071
alditol metabolic process	3	0.05	GO:0019400
response to abiotic stimulus	11	0.01	GO:0009628
phosphate metabolic process	21	0.01	GO:0006796
phosphorus metabolic process	21	0.01	GO:0006793
phosphorylation	15	0.05	GO:0016310
regulation of cytoskeleton organization	5	0.02	GO:0051493
regulation of organelle organization	7	0.02	GO:0033043
membrane organization	13	0.03	GO:0016044
membrane invagination	11	0.03	GO:0010324
endocytosis	11	0.03	GO:0006897
transmission of nerve impulse	12	0.00	GO:0019226
synaptic transmission	10	0.01	GO:0007268
cell–cell signaling	10	0.03	GO:0007267
synaptic vesicle endocytosis	4	0.03	GO:0048488

GO—gene ontology.

**Table 4:** Molecular function groups identified by the functional annotation clustering analysis with |fold change| ≥ 4 and 2 (DAVID functional analysis).

**|Fold Change| ≥ 4 (DAVID Functional Analysis)**			
**GO Term**	**Gene Count**	***p*-Value**	**GO Accession**
ATPase activity, coupled to transmembrane movement of substances	3	0.04	GO:0042626
ATPase activity, coupled to movement of substances	3	0.04	GO:0043492
hydrolase activity, acting on acid anhydrides, catalyzing transmembrane movement of substances	3	0.04	GO:0016820
primary active transmembrane transporter activity	3	0.05	GO:0015399
P–P-bond-hydrolysis-driven transmembrane transporter activity	3	0.05	GO:0015405
hydrogen ion transmembrane transporter activity	3	0.02	GO:0015078
monovalent inorganic cation transmembrane transporter activity	3	0.02	GO:0015077
inorganic cation transmembrane transporter activity	3	0.03	GO:0022890
**|Fold Change| ≥ 2 (DAVID Functional Analysis)**			
**GO Term**	**Gene Count**	***p*-Value**	**GO Accession**
transition metal ion binding	42	0.01	GO:0046914
metal ion binding	49	0.02	GO:0046872
cation binding	50	0.02	GO:0043169
ion binding	50	0.03	GO:0043167
cytoskeletal protein binding	14	7.58 × 10^−4^	GO:0008092
actin cytoskeleton organization	10	0.00	GO:0030036
actin filament-based process	10	0.00	GO:0030029
actin binding	9	0.00	GO:0003779
cytoskeleton organization	17	0.02	GO:0007010
nucleotide binding	37	0.02	GO:0000166
ATP binding	24	0.05	GO:0005524
adenyl ribonucleotide binding	24	0.05	GO:0032559
cytoskeletal protein binding	14	7.58 × 10^−4^	GO:0008092
microtubule binding	6	0.01	GO:0008017
tubulin binding	6	0.01	GO:0015631
hydrolase activity, acting on acid anhydrides, catalyzing transmembrane movement of substances	9	0.01	GO:0016820
primary active transmembrane transporter activity	9	0.02	GO:0015399
P–P-bond-hydrolysis-driven transmembrane transporter activity	9	0.02	GO:0015405
ATPase activity, coupled to movement of substances	8	0.03	GO:0043492
ATPase activity, coupled to transmembrane movement of substances	8	0.03	GO:0042626

GO—gene ontology.

## Data Availability

The data that support the findings of this study are available from the corresponding author upon reasonable request.
